# Red yeast rice ameliorates non-alcoholic fatty liver disease through inhibiting lipid synthesis and NF-κB/NLRP3 inflammasome-mediated hepatic inflammation in mice

**DOI:** 10.1186/s13020-022-00573-z

**Published:** 2022-01-25

**Authors:** Jian Zou, Chunyan Yan, Jian-Bo Wan

**Affiliations:** 1 State Key Laboratory of Quality Research in Chinese Medicine, Institute of Chinese Medical Sciences, University of Macau, Macao SAR Taipa, China; 2 School of Clinical Pharmacy, Guangdong Pharmaceutical University, Guangzhou, 510006 China

**Keywords:** NAFLD, NF-κB, NLRP3 inflammasome, Red yeast rice, Lipid synthesis

## Abstract

**Background:**

Red yeast rice (RYR), a nutraceutical with a profound cholesterol-lowering effect, was found to attenuate non-alcoholic fatty liver disease (NAFLD) in mice. Despite monacolin K in RYR being a specific inhibitor of hydroxymethylglutaryl-coenzyme A reductase (HMCGR), the mechanisms underlying the protective effects of RYR against NAFLD are not fully elucidated.

**Methods:**

Using a mouse model of high-fat diet (HFD) feeding and a cellular model of HepG2 cells challenged by lipopolysaccharide (LPS) and palmitic acid (PA), the possible molecular mechanisms were exploited in the aspects of NF-κB/NLRP3 inflammasome and mTORC1-SREBPs signaling pathways by examining the relevant gene/protein expressions. Subsequently, the correlation between these two signals was also verified using cellular experiments.

**Results:**

RYR ameliorated lipid accumulation and hepatic inflammation in vivo and in vitro. RYR improved lipid metabolism through modulating mTORC1-SREBPs and their target genes related to triglyceride and cholesterol synthesis. Furthermore, RYR suppressed hepatic inflammation by inhibiting the NF-κB/NLRP3 inflammasome signaling. Interestingly, the treatment with RYR or MCC950, a specific NLRP3 inhibitor, resulted in the reduced lipid accumulation in HepG2 cells challenged by LPS plus PA, suggesting that the inhibitory effects of RYR on NLRP3 inflammasome-mediated hepatic inflammation may partially, in turn, contribute to the lipid-lowering effect of RYR.

**Conclusions:**

The modulation of NF-κB/NLRP3 inflammasome and lipid synthesis may contribute to the ameliorative effects of RYR against HFD-induced NAFLD.

**Supplementary Information:**

The online version contains supplementary material available at 10.1186/s13020-022-00573-z.

## Introduction

Non-alcoholic fatty liver disease (NAFLD) has become a leading cause of chronic liver diseases in developed countries, involving clinical manifestations from simple hepatic steatosis to steatohepatitis [1]. The presence of non-alcoholic steatohepatitis (NASH) always makes the liver more susceptible to fibrosis, cirrhosis, and hepatocarcinoma [2]. To date, the exact mechanisms underlying NAFLD are not fully elucidated, but the “two-hit” hypothesis has played a crucial role in understanding the pathogenesis of NAFLD. The first hit involves hepatic lipid accumulation, the second hit accompanies by inflammation in the liver [3].

The nucleotide-binding oligomerization domain (NOD)-like receptor family pyrin domain containing 3 (NLRP3) belongs to the pattern recognition receptors (PPRs) family, which is consist of NLRP3 protein, apoptosis-associated speck-like protein containing a CARD (ASC), and cysteinyl aspartate specific proteinase-1 (caspase-1), the complexes of which is termed as NLRP3 inflammasome [4]. A two-step process is required in the activation of NLRP3 inflammasome. Firstly, the activation of Toll-like receptor (TLR) by its ligands, such as lipopolysaccharide (LPS), leads to transcriptional expression of pro-IL-1β, pro-IL-18, and inflammasome components. Secondly, the activation of NLRP3 inflammasome upon the sensation of pathogen-associated molecular patterns (PAMPs) or damage-associated molecular patterns (DAMPs) causes the cleavage of caspase-1, which subsequently cleaves pro-IL-1β and pro-IL-18 into their active forms, IL-1β and IL-18 [5, 6]. NLRP3 inflammasome was reported to be associated with the histologic severity of NAFLD in patients [7], and NLRP3 plays a crucial role in the pathogenesis of NASH induced by methionine choline-deficient diet (MCD) [8], high-fat diet (HFD) [9], and high fat-cholesterol-sugar diet (HF-HC-HSD) [10, 11]. NLRP3 inflammasome activation correlated with liver steatosis, inflammation, fibrosis, and tumorigenesis [10, 12, 13]. The blockage of NLRP3 inflammasome or caspase-1 alleviated liver steatosis, inflammation, and fibrosis in NAFLD [14–17]. Moreover, mice deficient in NLRP3 inflammasome failed to develop HFD-induced obesity, accompanied by the reduced triglyceride contents [18]. It is noteworthy that the activated caspase-1 participates in the proteolytic activation of sterol regulatory element-binding proteins (SREBPs), a transcription factor family responsible for modulating lipid homeostasis in mammals, which are regulated by peroxisome proliferator-activated receptor α (PPARα) [19–21]. Moreover, the mammalian target of rapamycin complex 1 (mTORC1) is essential for SREBPs activity, and rapamycin, a mTORC1 inhibitor, impairs the nuclear accumulation of SREBPs and downregulates the expression of lipogenic genes [22–25]. In addition, IL-1β facilitates lipid synthesis by upregulating diacylglycerol acyltransferase 2 (DGAT2), an essential enzyme in triglyceride synthesis [26], and inhibits fatty acid β-oxidation by regulating PPARα [27]. This evidence has tightly linked inflammasome activation with lipid metabolism in NAFLD, suggesting the involvement of NLRP3 inflammasome in driving the progression of NAFLD and the significance of blocking NLRP3 inflammasome for the treatment of NAFLD.

Red Yeast Rice (RYR) is the product of rice fermented with *Monascus purpureus* of the Aspergillaceae family. RYR was reported to be the most effective cholesterol-lowering nutraceuticals in the clinic [28, 29]. This efficacy is mainly attributed to monacolin K, called lovastatin, a selective inhibitor of hydroxymethylglutaryl-coenzyme A reductase (HMGCR) [29, 30]. In recent years, an increasing number of studies have shown that RYR achieved profound therapeutic effects in managing hypercholesterolemia [31], hyperlipidemia, NAFLD, and other metabolic disorders [32] in experimental studies and clinical trials. Studies have showed that statins also exert anti-inflammatory activity [33, 34]. The statins inhibit TLRs and NLRP3 inflammasome to treat atherosclerosis and other cardiovascular diseases (CVD) [33–35]. However, the exact mechanisms of RYR against NAFLD remains to be fully elucidated. In this study, therefore, the potential effect of RYR on HFD-induced lipid accumulation and hepatic inflammation was investigated in vivo and in vitro*.* Furthermore, their molecular mechanisms, in the aspect of NF-κB/NLRP3 inflammasome and lipid synthesis, were elucidated.

## Materials and methods

### Animals and treatments

C57BL/6J mice were purchased from Animal Research Core, University of Macau. After a week of adaption, male C57BL/6J mice (7-week-old) were fed a control diet (CON, D12450J, n = 6), a HFD (D12492, n = 10), and an HFD supplemented with RYR powder (HFD+RYR, n = 10, Hep Biotech Co., Ltd, Ningbo, China) for 16 weeks. The HFD+RYR diet, containing 5.1 g/kg RYR powder that consists of 3% of Monacolin K, provided the mice with RYR at a daily dose of 11.22 mg/kg body weight. The diet composition of each group is shown in Additional file 1: Table S1. Food intake was monitored three times a week and shown in Additional file 1: Figure S2. On the last day of the 16th week, mice were anesthetized. Serum samples were isolated and subsequently stored at − 80 °C. The entire liver tissue was immediately harvested, rinsed with ice-cold PBS, subsequently snap-frozen, and stored at − 80 °C for further analysis. The epididymal fat was also collected and weighted. All animal experiments were approved by the Animal Research Ethics Committee (Approval No. AEC-13-002-1), Institute of Chinese Medical Sciences, University of Macau.

### Biochemical and histological analysis

Serum levels of alanine aminotransferase (ALT) and aspartate aminotransferase (AST), total triglycerides (TG), and cholesterol (TC) were detected using their corresponding commercial kits (Nanjing Jiancheng Bioengineering Institute, Nanjing, China) according to the manufacturer’s protocols. IL-1β level in the cell culture medium was measured using Human ELISA Standard kits according to the manufacturer’s instruction (Cloud-Clone, Wuhan, China). H&E staining was conducted to determine the histological changes of the liver as previously described [36].

### Measurement of lipid accumulation in the liver and HepG2 cells

Oil Red O staining and lipid quantification kits were carried out to measure the lipid contents in the liver and the cells. For Oil Red O staining, liver cryostat section (8 μm) or HepG2 cells were fixed and then were stained with Oil Red O (Solarbio, Beijing, China) according to the standard protocol. For hepatic lipid quantification, approximately 100 mg of the liver tissue was homogenized in 0.2 mL of normal saline solution, and then hepatic lipids were extracted by 0.8 mL of chloroform/methanol solution (2:1, *v*/*v*). After centrifugation, the chloroform layer was collected to measure TC and TG using corresponding kits. For lipid quantification in cell lysates, cells were rinsed with ice-cold PBS solution twice and then lysed with 2% Triton X-100 in PBS, the lysate of which was subsequently subjected to TC and TG detection.

### Immunofluorescence staining

Immunofluorescence analysis of CD11b and F4/80 in the liver was conducted as described [37]. Briefly, cryostat section (8 μm) of the liver were fixed and washed with PBS. Following 1 h of blocking in 2% PBST containing 5% goat serum, the sections were incubated with rabbit anti-CD11b (1:100, Abcam) or rabbit anti-F4/80 antibody (1:50, Abcam) overnight at 4 ℃, the slices were incubated with Alexa Fluor® 488 goat anti-rabbit IgG (1:200, Abcam) for 2 h at 37 °C in the dark. After staining nuclei with DAPI, the slices were visualized and photographed under a confocal laser scanning microscope.

### Cell culture and treatment

For cell experiments, 270 mg of RYR powder was immersed and extracted with the mixture of water, ethanol, and DMSO (8:1:1, *v/v/v*, 2 mL) at room temperature for 2 h, the resulting solution was centrifuged, and the supernatant was filtered using 0.22 μm filter membrane (Millipore, USA) [38]. HepG2 cells obtained from the American Type Culture Collection (Manassas, VA, USA) were cultivated in RPMI 1640 medium (Hyclone, Logan, UT). To develop a cellular model of lipid accumulation, cells were stimulated with 20 µg/mL LPS (Solarbio, Beijing, China) for 3 h followed by incubation with or without RYR (337 µg/mL and 674 µg/mL), lovastatin (50 µM, Innochem, Beijing, China) or MCC950 (10 µM, Selleck, USA) for 24 h, subsequently, the cells were subject to 0.25 mM palmitate acid (PA) (Sigma, St. Louis, MO, USA)/1% BSA (Solarbio, Beijing, China) for another 24 h. The stock solution of 5 mM PA/5% BSA solution was prepared as described [39].

### RT-PCR

Total RNA of liver tissues was extracted using TRIZOL reagent (Invitrogen, Carlsbad, CA, USA) and transcribed into cDNA. qRT-PCR analysis was conducted in an ABI PRISM 7500 FAST Real-TIME PCR System (ABI, Vernon, CA, USA) using TIANGEN Talent qPCR PreMix (FP209-02, Tiangen). GAPDH was used to normalize mRNA levels. The sequences of gene-specific primers were shown in Additional file 1: Table S2.

### Immunoblot assay

Liver tissue from the same lobe was homogenized, and total protein was extracted using RIPA lysis buffer. Immunoblot assay was conducted as the standard protocol. GAPDH or β-actin was used as the internal control. The primary and secondary antibodies used in immunoblot analysis were listed in Additional file 1: Table S3.

### Statistical analysis

Statistical analyses were performed using a GraphPad Prism 8.0 (San Diego, CA, USA). The independent t-test determined the significance of between-group differences,and One-way analysis of variance (ANOVA) followed by Tukey’s post hoc test was used to evaluate the difference across multiple groups. All data were presented as Means ± SEM, and *p* < 0.05 was considered a significant difference.

## Results

### RYR ameliorates hepatic lipid accumulation and liver injury in HFD-fed mice

**Fig. 1:**
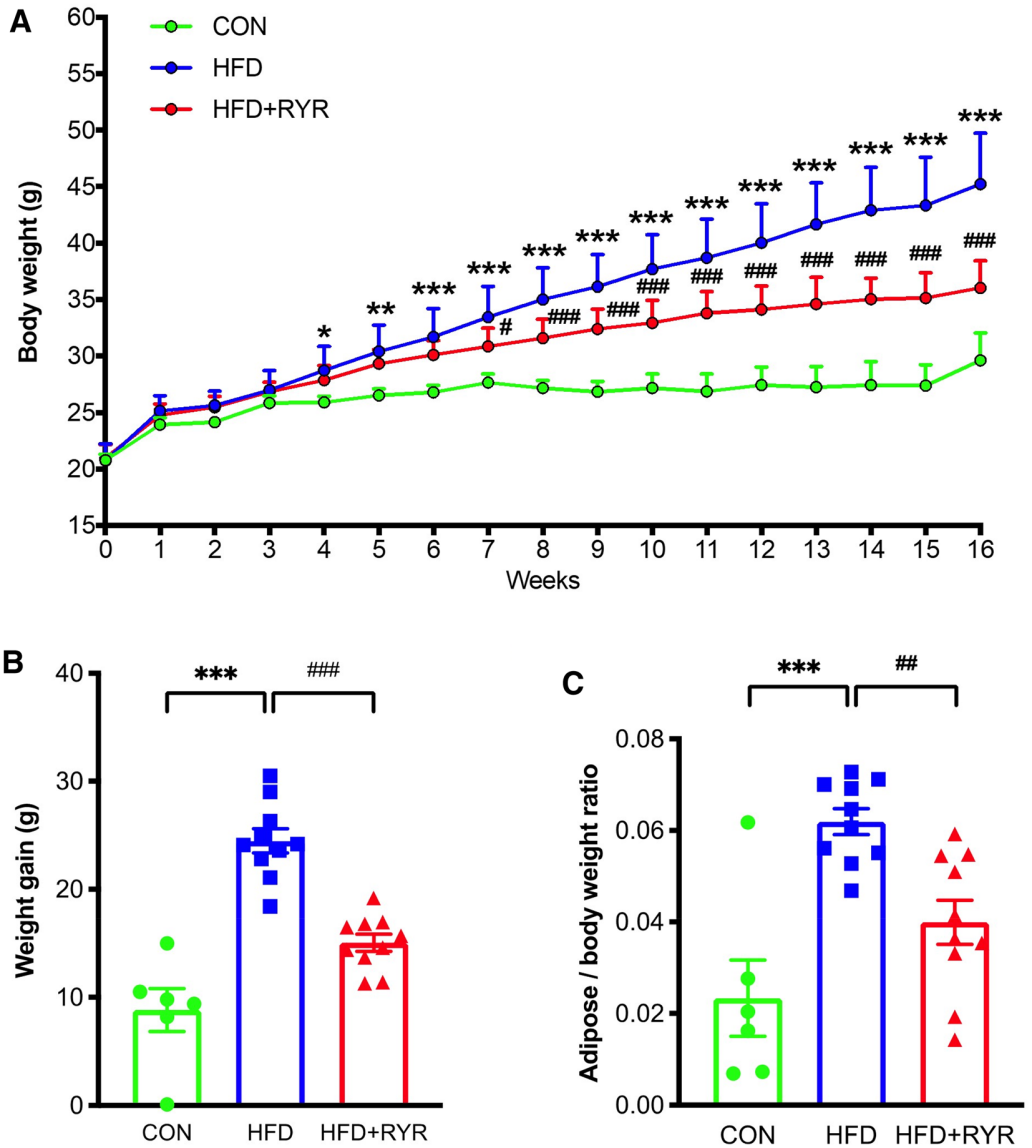
Effect of RYR on body weight, weight gain, and the ratio of adipose to body weight in HFD-fed mice. **A** Body weight curve. **B** weight gain. F(2, 23) = 39.8. **C** The ratio of adipose to body weight. F(2, 23) = 13.9, data shown are individual values with means ± SEM (n = 6–10). *p<0.05, **p<0.01, ***p<0.001 vs. control group. ^#^*p *< 0.05, ^###^*p *< 0.001 vs. HFD-fed group

**Fig. 2:**
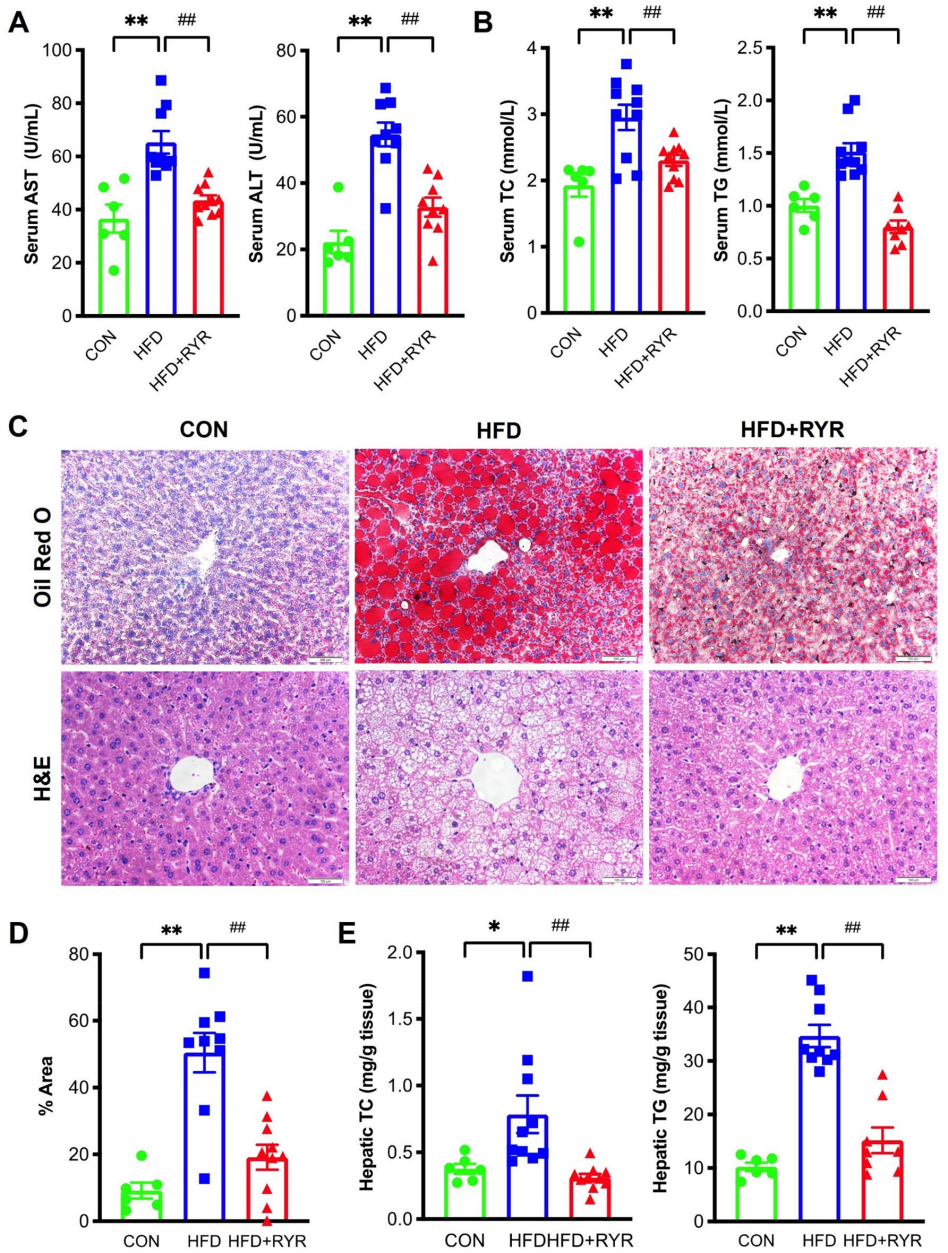
RYR attenuates liver injury and hepatic steatosis in HFD-fed mice.** A** Serum AST and ALT levels. **B** Serum TC and TG levels. **C** The representative histology of Oil Red O (scale bar, 200 μm) and H&E staining (scale bar, 100 μm). **D** Quantification of the positive area in Red Oil O staining. F(2,22) = 20.8. **E** Hepatic TC and TG levels. Data shown are individual values with means ± SEM (n = 6–10). *p < 0.05, **p < 0.01, vs. control group. ^##^*p *< 0.01 vs. HFD-fed group

Compared with the control diet (CON) group, the dramatic increases in body weight, weight gain, and adipose-to-body weight ratio were observed in the HFD group, which were significantly reversed in the HFD+RYR group (Fig. 1). There is no significant difference in the liver-to-body weight ratio across three groups (Additional file 1: Fig. S1). HFD-induced liver injury was examined using biochemical and histological analysis. HFD feeding for 16 weeks significantly elevated ALT and AST serum levels by 2.46-fold and 1.78-fold, respectively. These elevations were significantly decreased by the supplementation with RYR (Fig. 2A). RYR also inhibited serum levels of TC and TG elevated by HFD (Fig. 2B). Furthermore, hepatic lipid accumulation was increased by HFD feeding and markedly decreased in the RYR-treated group, as measured by both oil red O and H&E staining (Fig. 2C). These results have been validated by quantifying positive areas in oil red O staining (Fig. 2D) and quantitative measurements of hepatic TC and TG levels (Fig. 2E). These data collectively indicate that hepatic lipid accumulation and liver injury induced by HFD is ameliorated with the supplement of RYR.

### RYR inhibits HFD-induced hepatic inflammation via inhibiting NF-κB/NLRP3 activation

**Fig. 3:**
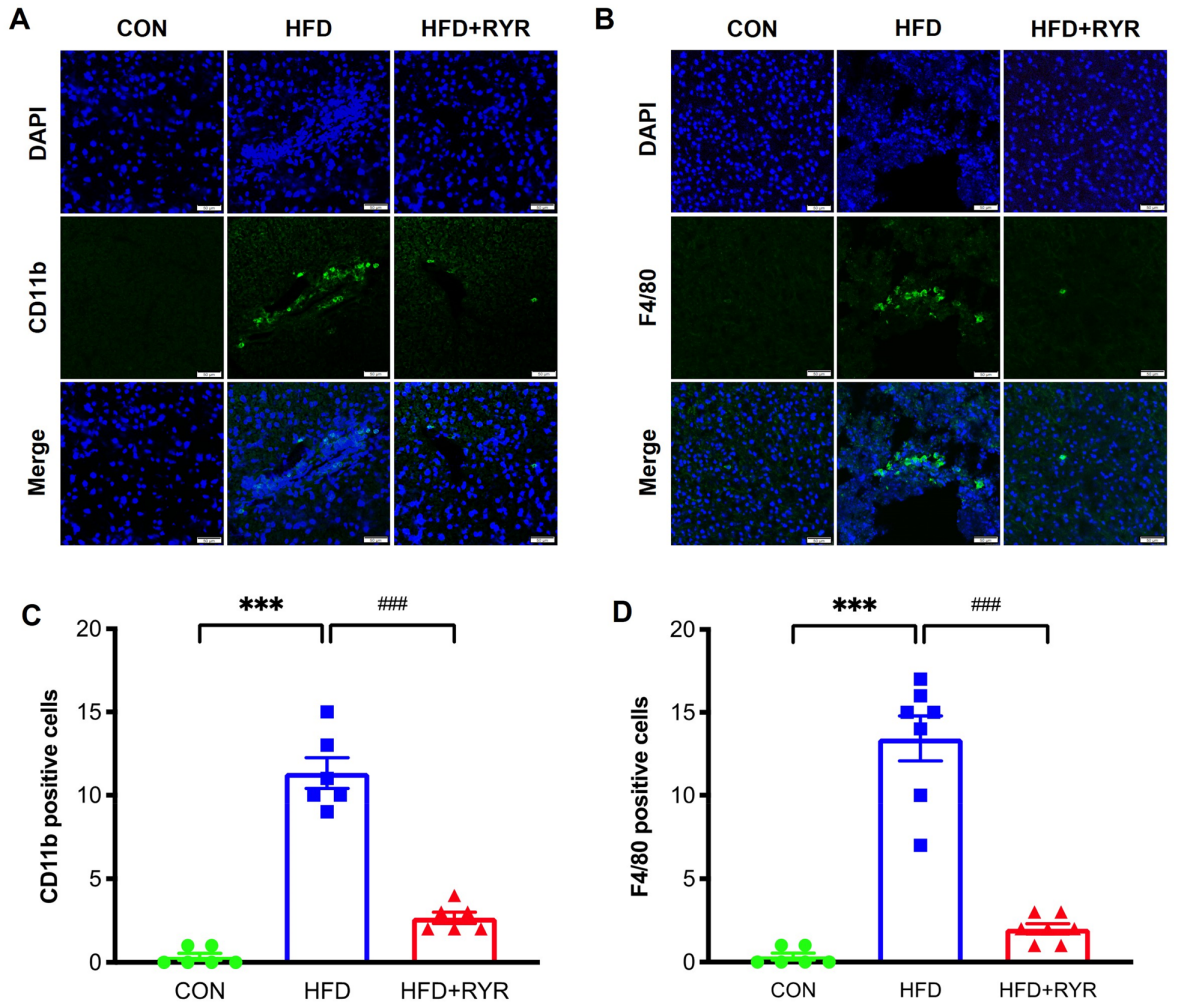
RYR inhibits hepatic inflammation in HFD-fed mice. **A**, **B** Immunofluorescent staining of liver sections (scale bar, 50 μm) for neutrophils (CD11b) and macrophages (F4/80), the nuclei were stained with DAPI (blue). **C**, **D** Quantification of CD11b- and F4/80-positive areas (n = 6–7 per group), each point represents the average number of positive cells in three randomly selected high power fields from each mouse). Data shown are individual values with means ± SEM. ***p < 0.001 vs. control group. ^###^*p *< 0.001 vs. HFD-fed group

To examine the possible effects of RYR on HFD-induced hepatic inflammation, the immunofluorescent staining of CD11b and F4/80, the specific monocyte and macrophage markers, in the liver tissue were examined. As shown in Fig. 3A, B, more CD11b- and F4/80-positive cell numbers were observed in the section of liver tissue from HFD-fed mice, over the CON group, RYR supplementation significantly decreased the infiltration of monocyte and macrophage in the liver. These visualized results were also confirmed by their quantitative analysis (Fig. 3C, D).

**Fig. 4:**
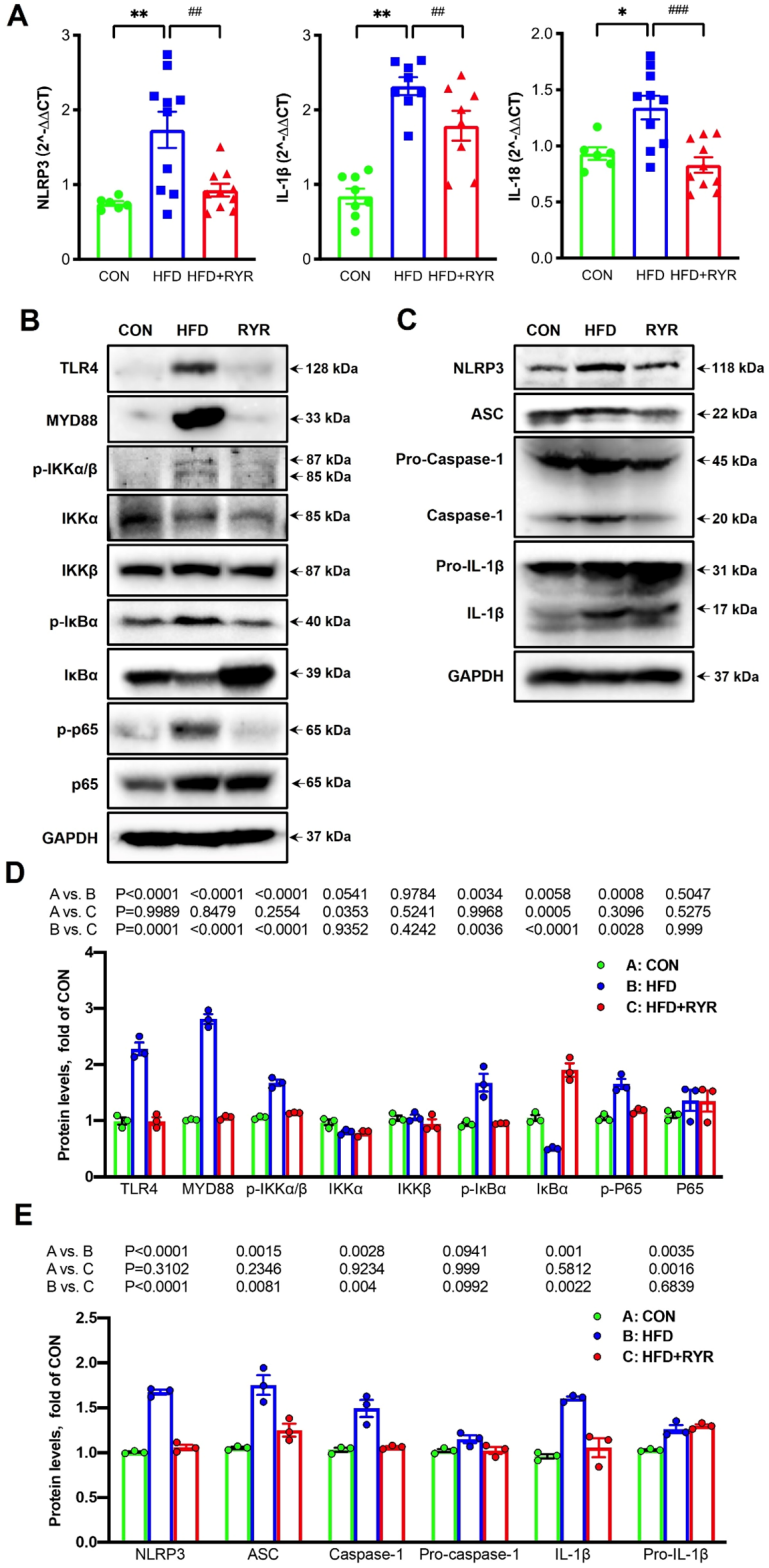
RYR ameliorates hepatic inflammation via inhibiting NF-κB/NLRP3 signaling in HFD-fed mice. **A** mRNA expressions of NLRP3, IL-1β and IL-18. **B** Immunoblot analysis of TLR4/NF-κB signaling in the liver, including TLR4, MYD88, p-IKKα/β, IKKα, IKKβ, p-IκBα, IκBα, p-p65, and p65. **C** Hepatic protein expressions of NLRP3, ASC, pro-caspase-1, caspase-1, pro-IL-1β and IL-1β. GAPDH was used as a control. **D**,**E** The densitometry analysis results of the Immunoblot images of the TLR4/NF-κB and NLRP3 inflammasome signaling. Data shown are individual values with means ± SEM (n = 3). **p < 0.01, vs. control group. ^##^*p *< 0.01, vs. HFD-fed group

Activation of NLRP3 inflammasome signaling plays a crucial role in HFD-induced hepatic inflammation [4, 40]. We next examined the transcriptional expressions of NLRP3, IL-1β, and IL-18 in the liver by qPCR. As shown in Fig. 4 A, compared to the CON group, the mRNA expressions of NLRP3, IL-1β and IL-18 were upregulated in HFD-fed mice, which were significantly reversed by supplement with RYR. The activation of NLRP3 inflammasome results in the maturation of caspase-1, which cleaves pro-cytokines, such as pro-IL-1β and pro-IL-18, into inflammatory cytokines. While the productions of pro-cytokines rely on NF-κB activation. As shown in Fig. 4B and D, HFD-fed mice had the remarkable increases in hepatic expressions of TLR4, and its downstream proteins, including MYD88, p-IKKα/β, p-IκBα, and p-p65, and a decrease in the expression of IκBα without changing the expressions of total IKKβ and p65, over CON group. These upregulated protein expressions were reversed by supplement with RYR. Meanwhile, the upregulated hepatic expressions of NLRP3, ASC, the active isoform of caspase-1, and mature IL-1β were observed in HFD-fed mice, compared to the CON group, but remarkably downregulated in the RYR-treated group (Fig. 4C, E). Collectively, our data indicate that NF-κB/NLRP3 signaling is implicated in the inhibitory effects of RYR on HFD-induced hepatic inflammation.

### RYR regulates triglyceride and cholesterol synthesis

**Fig. 5:**
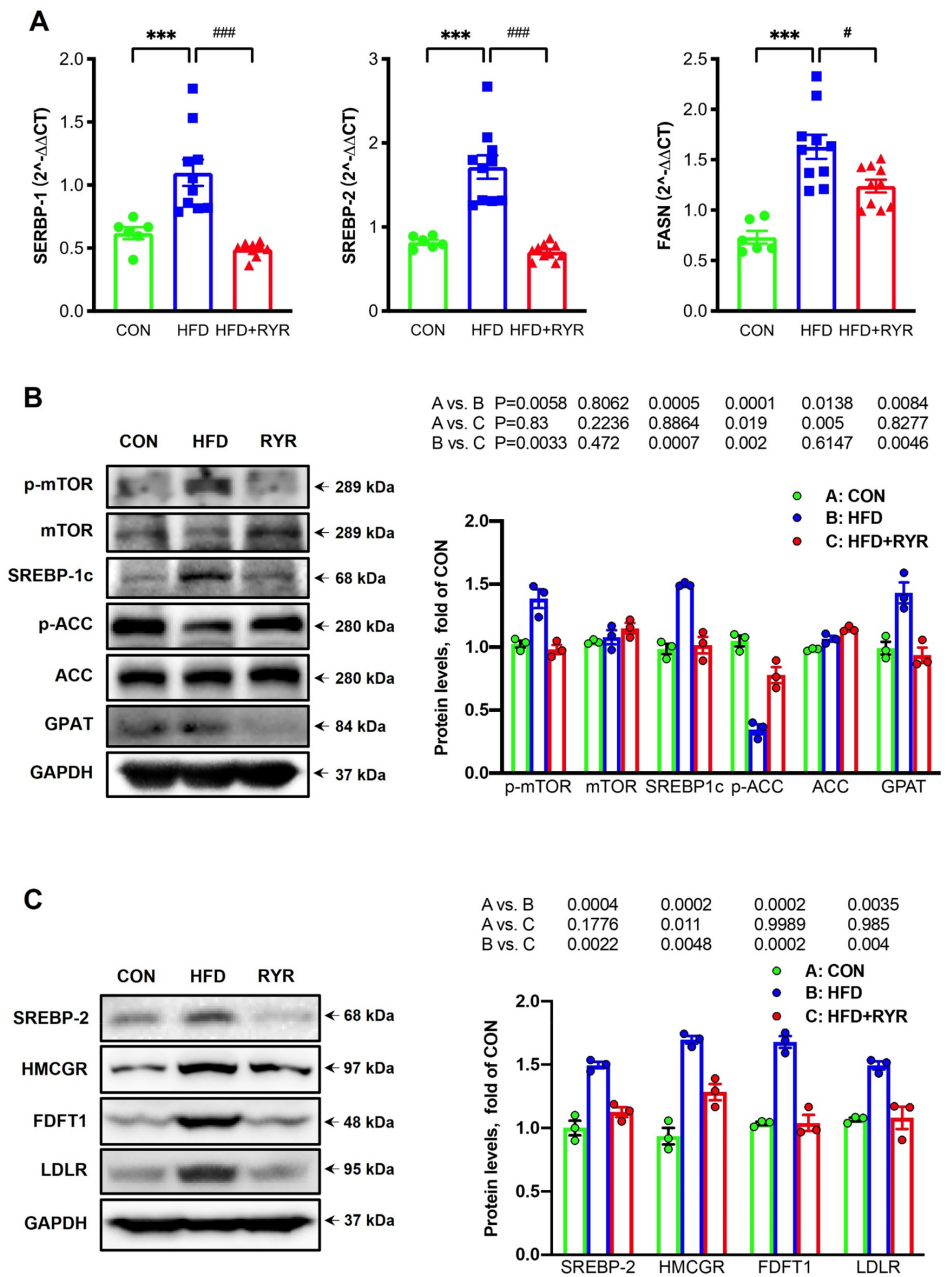
RYR reverses lipid synthesis via inhibiting mTORC1-SREBPs signaling in HFD-fed mice.** A** qPCR analysis of genes involved in lipogenesis-related genes in the liver, including SREBP-1, SREBP-2 and FASN. **B** Immunoblot analysis of mTOR, SREBP-1c, ACC and GPAT proteins related to lipogenesis in the liver (left) and their densitometric analysis (right, n = 3). **C** Hepatic expressions of proteins related to cholesterol metabolism, including SREBP-2, HMCGR, FDFT1, and LDLR (left) and their densitometric analysis results (right, n = 3). GAPDH was used as a control

To understand the molecular mechanisms underlying the protective effects of RYR against HFD-induced hepatic lipid accumulation, the mRNA expression of SREBPs were examined by qPCR. As shown in Fig. 5A, HFD feeding significant upregulated the mRNA expression of SREBP-1 and SREBP-2, both of which were dramatically inhibited by the treatment of RYR. In addition, we also examined the mRNA expression of fatty acid synthase (FASN), an enzyme responsible for the final step of palmitic acid synthesis in de novo lipogenesis. HFD induced significant elevation of FASN, which was also down-regulated by RYR. Furthermore, the protein expressions of genes related to triglyceride and cholesterol synthesis in the liver were further examined by immunoblot analysis. As shown in Fig. 5B, HFD significantly induced the hepatic expressions of p-mTOR, SREBP-1c and glycerol-3-phosphate acyltransferase (GPAT), the rate-limiting enzyme responsible for the synthesis of glycerophospholipids and triglycerides, which were remarkably reversed by the supplement with RYR. At the same time, HFD de-phosphorylated and activated acetyl-CoA carboxylase (ACC), the rate-limiting enzyme in de novo lipogenesis process, while the treatment of RYR reversed this change without affecting the expression of total ACC. As expected, hepatic expressions of proteins related to cholesterol synthesis, including SREBP-2, HMCGR, farnesyl-diphosphate farnesyltransferase 1(FDFT1), and LDL receptor (LDLR), were upregulated by HFD feeding, which was markedly downregulated by the supplement with RYR (Fig. 5C). Collectively, our data indicate that RYR regulates lipid metabolism via the inhibition of mTORC1-SREBPs.

### RYR reduces lipid accumulation and IL-1β secretion induced by LPS plus PA in HepG2 cells

**Fig. 6:**
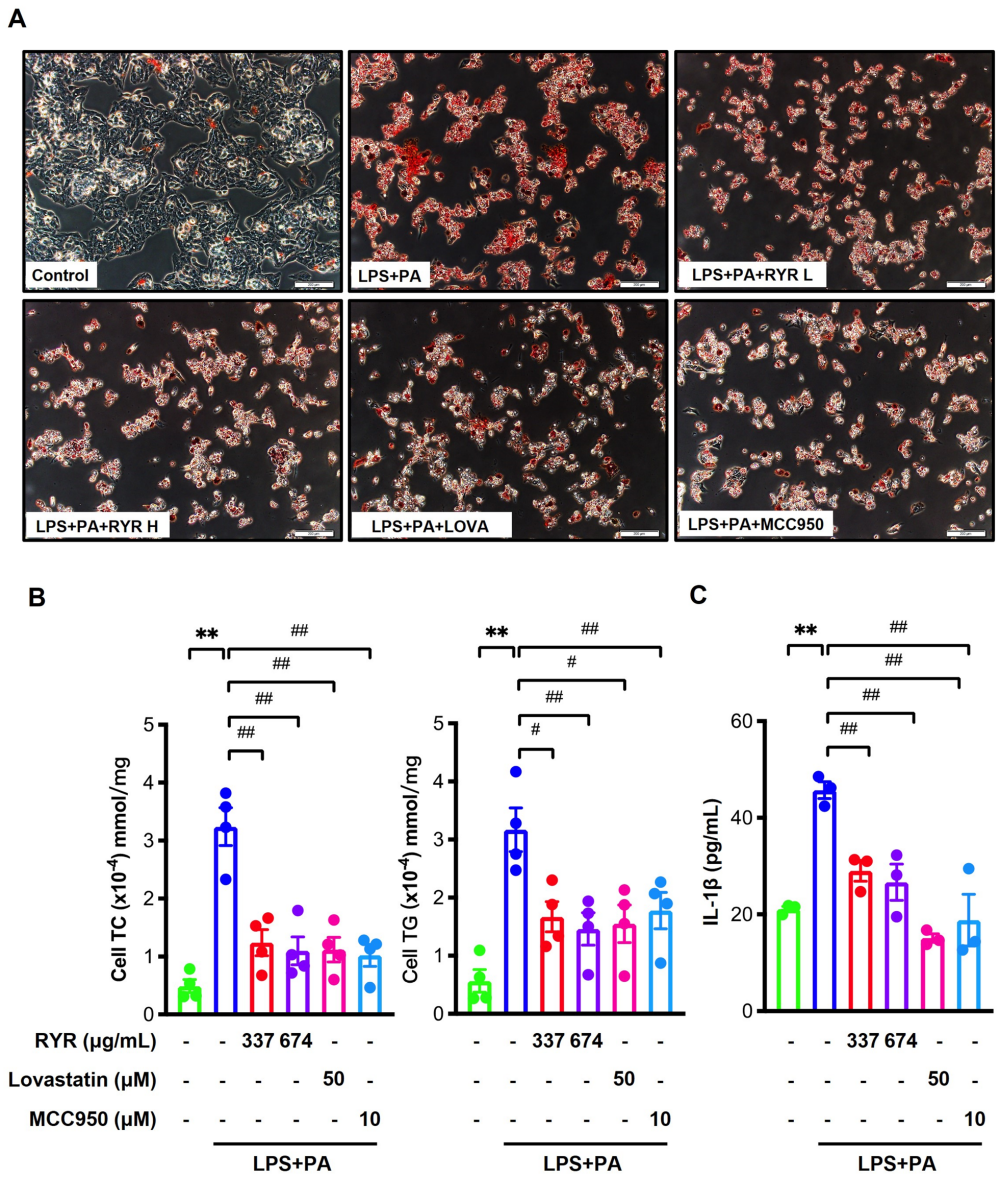
RYR inhibits lipid accumulation and IL-1β secretion induced by LPS plus PA in HepG2 cells. **A** Cellular lipid droplets were measured by Oil Red O staining. **B** Cellular TG and TC levels (n = 4). **C** IL-1β in cell culture medium (n = 3). Data shown are individual values with means ± SEM. HepG2 cells were exposed to PBS plus 0.25 mM NaOH/1% BSA alone, LPS plus 0.25 mM PA/1% BSA, LPS plus 0.25 mM PA/1% BSA pretreated with 337 µg/mL, 674 µg/mL RYR, 50 µM lovastatin or 10 µM MCC950, respectively. **p<0.01, vs. control group. ^#^*p*<0.05, ^##^*p*<0.01 vs. LPS plus PA group

To develop a cell culture model of lipid overload, HepG2 cells were stimulated with LPS plus PA for 24 h. Lovastatin was used as a positive control. After being challenged by LPS plus PA, the massive lipid accumulation was observed as shown by Oil Red O staining, which was remarkably relieved by RYR treatment at 337 µg/mL (low dose) and 674 µg/mL (high dose), lovastatin (50 µM) or MCC950 (10 µM), a specific inhibitor of NLRP3 inflammasome (Fig. 6A). Cell lysates were subsequently subjected to the quantification of TC and TG levels. Consistently, the cellular TC and TG levels were significantly increased upon the co-stimulation with LPS plus PA but dramatically reduced by the treatment of RYR, lovastatin, or MCC950 (Fig. 6B). These data indicate that the inhibition of NLRP3 inflammasome largely contributes to the lipid-lowering effects of RYR. Moreover, the stimulation of LPS plus PA resulted in a significant increase of IL-1β in the cell culture supernatant, which was also reduced upon the treatment with RYR, lovastatin, or MCC950 (Fig. 6C).

### RYR inhibits NF-κB/NLRP3 inflammasome pathway and SREBPs signaling in HepG2 cells

**Fig. 7:**
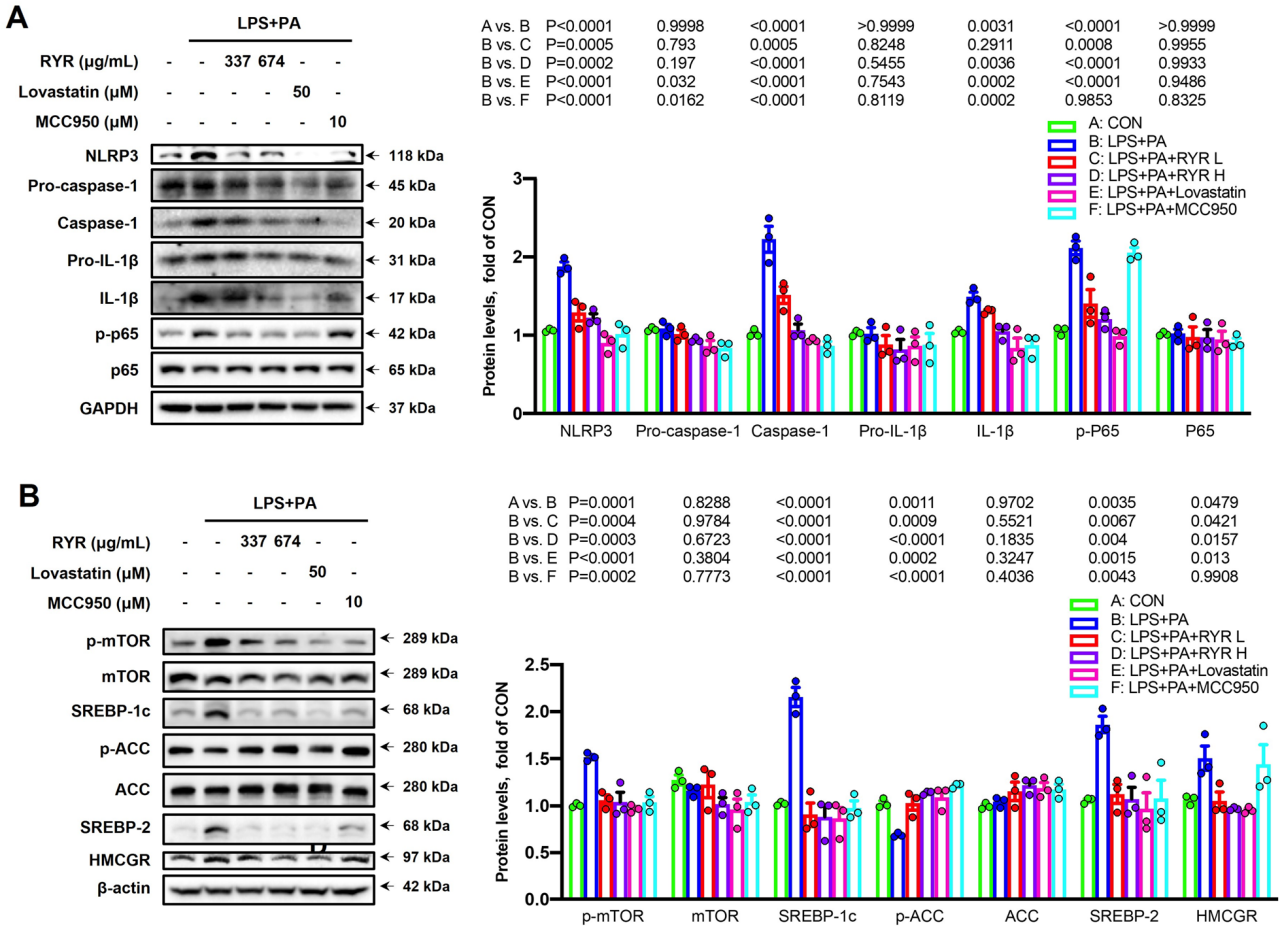
RYR ameliorates cellular inflammation in HepG2 cells via inhibiting NLRP3/NF-κB signaling. **A** Immunoblot analysis of NLRP3, (pro)-caspase-1, (pro)-IL-1β, (p-)p65 in cell lysates (left) and their densitometric analysis (right, n = 3). **B** Hepatic protein expressions of SREBP-2, HMCGR, SREBP-1c, and ACC in cell lysates (left) and their densitometric analysis results (right, n = 3). β-actin or GAPDH was used as a control

Immunoblot analysis revealed that the co-stimulation of LPS plus PA activated NLRP3 inflammasome in HepG2 cells, as evidenced by the upregulated protein expressions of NLRP3, caspase-1, and IL-1β in cell lysates of HepG2, which were dramatically downregulated with the treatment of RYR, lovastatin, or MCC950 (Fig. 7A, C). Furthermore, the co-stimulation of LPS and PA provoked NF-κB signaling, the increased protein expression of phosphorylation of p65 in cell lysates was observed, which were decreased by either RYR or lovastatin, but not by MCC950, an NLRP3 inhibitor (Fig. 7A, C). Additionally, the protein expressions of SREBP-2 and SREBP-1c were also examined, the immunoblot analysis results showed that the stimulation of LPS and PA-induced the activation of p-mTOR, SREBP-1c and p-ACC as well as SREBP-2 and HMCGR, while the treatment with RYR or lovastatin significantly decreased their overexpression (Fig. 7B and D). The in vitro results were consistent with the in vivo results that RYR inhibits hepatic lipid deposition through modulating mTORC1-SREBPs. Most importantly, the inhibitory effect of MCC950 on cellular lipid accumulation, p-mTOR, and SREBPs induced by LPS plus PA suggested that the lipid-lowering effect of RYR was at least partially mediated through inhibiting the NF-κB and NLRP3 signaling pathway.

## Discussion

NAFLD has posed a significant public health burden, but the satisfactory therapeutic interventions for the management of NAFLD are limited [40]. Statins are competitive inhibitors of HMCGR, a rate-limiting enzyme in cholesterol synthesis [41]. In addition to the lipid-lowering effects, statins also possess anti-inflammatory effects, and it was reported that the inhibitory effects of statins on TLR4/NF-κB and NLRP3 inflammasome pathways contributed to the treatment of atherosclerosis and other inflammation-driven diseases [33–35]. RYR, whose main active ingredient is lovastatin, had profoundly beneficial effects in treating hyperlipidemia in numerous experimental studies and placebo-controlled clinical trials [42–46]. Although several previous studies have examined the beneficial effects of RYR in experimental NAFLD and NASH models [32, 47], the underlying mechanism of RYR against NAFLD is not fully elucidated. In this study, our data indicate that RYR ameliorates HFD-induced hepatic steatosis and inflammation by inhibiting NF-κB/NLRP3 inflammasome signaling and SREBP pathways in vivo and in vitro.

In NAFLD, the dysregulated lipid environment in the liver leads to the accumulation of harmful lipids within hepatocytes, which not only causes organelle dysfunction or cellular death but also serves as DAMPs for NLRP3 inflammasome [4, 48], the gut-derived LPS also provides the priming step for NLRP3 inflammasome activation [4]. Indeed, in this study, NLRP3 inflammasome is activated in mice after HFD feeding for 16 weeks as well as in HepG2 cells challenged with LPS plus PA, as demonstrated by increased expression of NF-κB signaling pathway and NLRP3, ASC, caspase-1, IL-1β in the liver, cell lysates as well as cell culture medium, while the treatment of RYR reduced the NF-κB signaling and NLRP3 activation in vivo and in vitro. Furthermore, the production of proinflammatory cytokines subsequently recruit neutrophils, monocytes, and macrophages into the liver, which further deteriorates liver damage through the release of cytotoxic proteases or reactive oxygen species (ROS) [47, 49], in this study, CD11b and F4/80 positive phagocytes infiltration to the liver were significantly elevated in HFD-fed mice, which were significantly reduced by the treatment of RYR.

Additionally, NLRP3 inflammasome plays a critical role in accelerating lipogenesis. The SREBPs mainly include three isoforms, SREBP-1a, SREBP-1c, and SREBP-2 [50]. SREBP-1a activates all SREBPs-responsive genes involved in fatty acids, triglycerides, and cholesterol. However, its expression presents at a low level in the liver [51, 52]. SREBP-1c is a predominant isoform of SREBP-1 in the liver, which preferentially activates the genes related to lipogenesis and triglyceride metabolisms [21]. While SREBP-2 mainly promotes the expression of LDLR and genes required for cholesterol synthesis, including HMGCR, and FDFT1 that also plays a critical role in cholesterol synthesis, and other related enzymes [50, 53]. Studies found that the activation of caspase-1 triggered S1P- and S2P-mediated SREBPs activation, thus promoting the expression of HMCGR and FASN, the target genes of SREBP-2 and SREBP-1c, respectively [19, 54]. Moreover, the inhibition of NLRP3 inflammasome with MCC950 suppressed the expression of SREBP-1c and its target genes FASN and ACC1 [55]. In addition, the secretion of IL-1β from Kupffer cells enhances lipid accumulation in a NASH model induced by choline-deficient amino acid-defined (CDAA) diet, while IL-1R^−/−^ mice exhibited reduced hepatic steatosis [26]. These findings suggest a strong correlation exists between NLRP3 inflammasome and lipid synthesis. Furthermore, the mTORC1 had been reported to be required for de novo lipogenesis in rat livers and cultured hepatocytes [56–58] Consistently, in this study, our results show that MCC950 not only dramatically decreased cellular TC and TG levels but also abolished the activation of mTOR, SREBP-1c, SREBP-2 and their target genes ACC and HMCGR induced by LPS plus PA in HepG2 cells. Moreover, RYR was found to abolish activation of NLRP3 inflammasome and upregulation of SREBPs, including SREBP-1c and SREBP-2 in the liver of HFD-fed mice and HepG2 cells, suggesting the possible contribution of NLRP3 inflammasome inhibitory effect to its lipid-lowering effect. Furthermore, the aberrant upregulation of LDLR in HFD-fed mice was also reduced by RYR, suggesting that RYR reduces cholesterol in the liver through inhibition of both cholesterol synthesis and uptake. Monacolin K, the competitive inhibitor of HMCGR, also directly contributes to the lipid-lowering effects of RYR. In addition, the RYR used in the current study contains 3% monacolin K, the effective dose, 337 and 674 µg/mL, contains approximately 10 µg/mL and 20 µg/mL monacolin K, respectively, which is equivalent to nearly 25 µM and 50 µM of monacolin K. Indeed, the protective effects of RYR at 674 µg/mL is comparable to lovastatin at 50 µM in vitro.

**Fig. 8:**
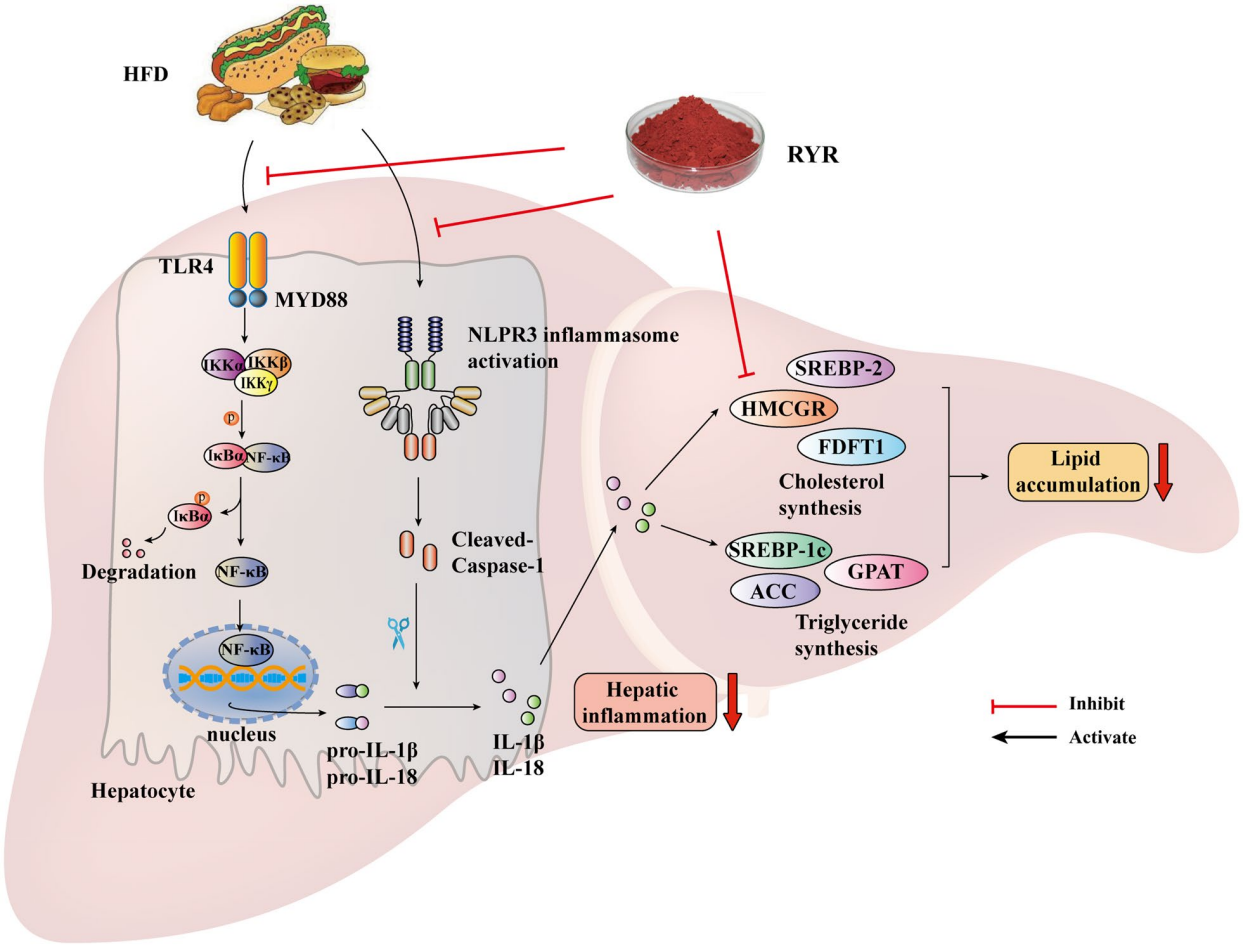
Schematic diagram of the potential mechanisms underlying the protective effects of RYR against NAFLD. RYR attenuates HFD-induced hepatic NF-κB signaling activation and then reduces the production of pro-IL-1β and pro-IL-18. Simultaneously, RYR blocks HFD-triggered activation of the NLRP3 inflammasome, resulting in decreased maturation and secretion of IL-1β and IL-18. The suppression of the aberrant inflammatory signaling by RYR regulates SREBP-1c, SREBP-2, and their target genes related to lipid synthesis. Meanwhile, monacolin K in RYR, acting as a competitive inhibitor of HMCGR, directly decreases the cholesterol synthesis in the liver

Collectively, our data clearly show that RYR protects against HFD-induced NAFLD by reducing steatosis and inflammation in the liver. These beneficial effects are associated with regulating lipid metabolism through modulating mTORC1-SREBPs and inhibiting hepatic inflammation via NF-κB/NLRP3 inflammasome signaling, the inhibitory effect of RYR on NLRP3 inflammasome might at least partially contribute to its lipid-lowering effect, as illustrated in Fig. 8.

## Supplementary Information

**Additional file 1: Fig. S1.** The ratio of liver to body weight across different groups; **Fig. S2.** The food intake of different groups during the feeding period; **Table S1.** Diet compositions of the CON, HFD and HFD+RYR groups; **Table S2.** Primer sequences of the genes used for quantitative real-time polymerase chain reaction; **Table S3.** Primary and secondary antibodies used in immunoblot analysis.

## Data Availability

All the data used to support the findings of this study are available from the corresponding author upon reasonable request.

## Abbreviations

ALT: Alanine aminotransferase
ASC: Apoptosis-associated speck-like protein containing a CARD
AST: Aspartate aminotransferase
Caspase-1: Cysteinyl aspartate specific proteinase-1
CDAA: Choline-deficient amino acid-defined
CVD: Cardiovascular diseases
DAMPs: Damage-associated molecular patterns
DGAT2: Diacylglycerol acyltransferase 2
DMSO: Dimethyl sulfoxide
FASN: Fatty acid synthase
FDFT1: Farnesyl-diphosphate farnesyltransferase 1
GPAT: Glycerol-3-phosphate acyltransferase
HFD: High-fat diet
HF-HC-HSD: High fat-cholesterol-sugar diet
HMGCR: Hydroxymethylglutaryl-coenzyme A reductase
H&E: Hematoxylin and eosin
LDLR: LDL receptor
LPS: Lipopolysaccharide
MCD: Methionine choline-deficient diet
NAFLD: Non-alcoholic fatty liver disease
NAFL: Non-alcoholic fatty liver
NASH: Non-alcoholic steatohepatitis
NLRP3: Nucleotide-binding oligomerization domain (NOD)-like receptor family pyrin domain containing 3
PA: Palmitic acid
PAMPs: Pathogen-associated molecular patterns
PBS: Phosphate-buffered saline
PPRs: Pattern recognition receptor
PVDF: Polyvinylidene fluoride membrane
ROS: Reactive oxygen species
RYR: Red yeast rice
SDS-PAGE: Sodium dodecyl-polyacrylamide gel electrophoresis
TC: Total cholesterol
TG: Triglycerides
TLR: Toll-like receptor
