# Supplementary material for: Red yeast rice ameliorates non-alcoholic fatty liver disease through inhibiting lipid synthesis and NF-κB/NLRP3 inflammasome-mediated hepatic inflammation in mice
Source: Chin Med. 2022 Jan 25;17:17. doi: 10.1186/s13020-022-00573-z (PMC8788078; doi:10.1186/s13020-022-00573-z)
Supplement: Supplementary file 1 — Additional file 1: Fig. S1. The ratio of liver to body weight across different groups; Fig. S2. The food intake of different groups during the feeding period; Table S1. Diet compositions of the CON, HFD and HFD+RYR groups; Table S2. Primer sequences of the genes used for quantitative real-time polymerase chain reaction; Table S3. Primary and secondary antibodies used in immunoblot analysis. [file 13020_2022_573_MOESM1_ESM.docx]

# Supplementary Materials

**Red yeast rice attenuates non-alcoholic fatty liver disease through inhibiting lipid synthesis and NF-κB/NLRP3 inflammasome-mediated hepatic inflammation**

**Fig. S1**. The ratio of liver to body weight across different groups

**Fig. S2**. The food intake of different groups during the feeding period

**Table S1. Diet compositions of the CON, HFD and HFD+RYR groups**

|  | **Control (CON, D12450J)** | |  | **High-fat diet (HFD, D12492)** | |  | **HFD+RYR** | |
| --- | --- | --- | --- | --- | --- | --- | --- | --- |
|  | **gm** | **kcal** |  | **gm** | **kcal** |  | **gm** | **kcal** |
| Casein, 80 Mesh | 200 | 800 |  | 200 | 800 |  | 200 | 800 |
| L-Cystine | 3 | 12 |  | 3 | 12 |  | 3 | 12 |
| Corn Starch | 506.2 | 2024.8 |  | 0 | 0 |  | 0 | 0 |
| Maltodextrin 10 | 125 | 500 |  | 125 | 500 |  | 125 | 484 |
| Sucrose | 68.8 | 275.2 |  | 68.8 | 275.2 |  | 68.8 | 275.2 |
| Cellulose, BW200 | 50 | 0 |  | 50 | 0 |  | 50 | 0 |
| Red Yeast Rice | 0 | 0 |  | 0 | 0 |  | 4 | 16 |
| Lard | 20 | 180 |  | 245 | 2205 |  | 245 | 2205 |
| Mineral Mix, S10026 | 10 | 0 |  | 10 | 0 |  | 10 | 0 |
| DiCalcium Phosphate | 13 | 0 |  | 13 | 0 |  | 13 | 0 |
| Calcium Carbonate | 5.5 | 0 |  | 5.5 | 0 |  | 5.5 | 0 |
| H_2_O | 16.5 | 0 |  | 16.5 | 0 |  | 16.5 | 0 |
| Vitamin Mix, V10001 | 10 | 40 |  | 10 | 40 |  | 10 | 40 |
| Choline Bitartrate | 2 | 0 |  | 2 | 0 |  | 2 | 0 |
| FD&C Blue Dye #1 | 0.01 | 0 |  | 0.05 | 0 |  | 0.05 | 0 |
| FD&C Yellow Dye | 0.04 | 0 |  | 0 | 0 |  | 0 | 0 |
| Total | 1055.05 | 4021 |  | 773.85 | 4057 |  | 773.85 | 4057 |

**Table S2. Primer sequences of the genes used for quantitative real-time polymerase chain reaction.**

| **Gene** | **Forward (5′ to 3′)** | **Reverse (5′ to 3′)** |
| --- | --- | --- |
| **NLRP3** | ATTACCCGCCCGAGAAAGG | TCGCAGCAAAGATCCACACAG |
| **IL-1β** | GAAATGCCACCTTTTGACAGTG | TGGATGCTCTCATCAGGACAG |
| **IL-18** | GACTCTTGCGTCAACTTCAAGG | CAGGCTGTCTTTTGTCAACGA |
| **FASN** | CACAGTGCTCAAAGGACATGCC | CACCAGGTGTAGTGCCTTCCTC |
| **SREBP1** | CGACTACATCCGCTTCTTGCAG | CCTCCATAGACACATCTGTGCC |
| **SREBP2** | AGAAAGAGCGGTGGAGTCCTTG | GAACTGCTGGAGAATGGTGAGG |
| **GAPDH** | TGACCTCAACTACATGGTCTACA | CTTCCCATTCTCGGCCTTG |

**Table S3. Primary and secondary antibodies used in immunoblot analysis.**

| **Primary antibody** | **Full name** | **Source** | **Dilution** | **Company** |
| --- | --- | --- | --- | --- |
| NLRP3 | NOD-, LRR- and pyrin domain-containing protein 3 | Rabbit | 1:1000 | Abcam |
| Caspase-1 | Cysteine-aspartic proteases-1 | Rabbit | 1:1000 | Abcam |
| IL-1β | InterLukin-1β | Rabbit | 1:1000 | Cell Signaling Technology |
| Cleaved-caspase-1 | Cleaved-InterLukin-1β | Rabbit | 1:1000 | Cell Signaling Technology |
| Cleaved-IL-1β | Cleaved Cysteine-aspartic proteases-1 | Rabbit | 1:1000 | Cell Signaling Technology |
| MYD88 | Myeloid differentiation primary response 88 (MYD88) | Rabbit | 1:1000 | Abcam |
| SREBP-1 | Sterol regulatory element binding protein 1 | Rabbit | 1:1000 | Abcam |
| SREBP-2 | Sterol regulatory element binding protein 2 | Rabbit | 1:1000 | Abcam |
| ACC | Acetyl-CoA carboxylase | Rabbit | 1:1000 | Cell Signaling Technology |
| p-ACC | Phosphorylated Acetyl-CoA carboxylase | Rabbit | 1:1000 | Cell Signaling Technology |
| mTOR | Mammalian target of rapamycin | Rabbit | 1:1000 | Protein Biotechnolgy |
| p-mTOR | Phosphorylated mammalian target of rapamycin | Rabbit | 1:1000 | Santa cruz biotechnology |
| HMCGR | 3-hydroxy-3-methyl-glutaryl-coenzyme A reductase | Rabbit | 1:1000 | Abcam |
| FDFT1 | Farnesyl-Diphosphate Farnesyltransferase 1 | Rabbit | 1:1000 | Abcam |
| GPAT | Glycerol-3-phosphate acyltransferase 1, mitochondrial | Rabbit | 1:1000 | Abcam |
| ASC | Apoptosis-associated speck-like protein containing a CARD | Rabbit | 1:1000 | Cell Signaling Technology |
| NF-κB pathway sample kit | / | Rabbit | 1:1000 | Cell Signaling Technology |
| IgG H&L (Alexa Fluor® 488) | / | Goat-anti-rabbit | 1:1000 | Abcam |
| HRP-conjugated IgG | / | Goat-anti-rabbit | 1:1000 | Cell Signaling Technology |
| GAPDH | Glyceraldehyde 3-phosphate dehydrogenase | Rabbit | 1:1000 | Abcam |
| β-actin | / | Rabbit | 1:1000 | Cell Signaling Technology |
